# {μ-2-[(3-Amino-2,2-dimethyl­prop­yl)imino­meth­yl]-6-meth­oxy­phenolato-1:2κ^5^ *O* ^1^,*O* ^6^:*N*,*N*′,*O* ^1^}{2-[(3-amino-2,2-dimethyl­prop­yl)imino­meth­yl]-6-meth­oxy­phenolato-1κ^3^ *N*,*N*′,*O* ^1^}-μ-azido-1:2κ^2^ *N*:*N*-azido-2κ*N*-methanol-2κ*O*-dinickel(II)

**DOI:** 10.1107/S1600536812029662

**Published:** 2012-07-04

**Authors:** Akbar Ghaemi, Saeed Rayati, Kazem Fayyazi, Seik Weng Ng, Edward R. T. Tiekink

**Affiliations:** a Department of Chemistry, Saveh Branch, Islamic Azad University, Saveh, Iran; b Department of Chemistry, K. N. Toosi University of Technology, PO Box 16315-1618, Tehran, Iran; c Department of Chemistry, University of Malaya, 50603 Kuala Lumpur, Malaysia; d Chemistry Department and Faculty of Science, King Abdulaziz University, PO Box 80203 Jeddah, Saudi Arabia

## Abstract

Two distinct coordination geometries are found in the binuclear title complex, [Ni_2_(C_13_H_19_N_2_O_2_)_2_(N_3_)_2_(CH_3_OH)], as one Schiff base ligand is penta­dentate, coordinating *via* the anti­cipated oxide O, imine N and amine N atoms (as for the second, tridentate, ligand) but the oxide O is bridging and coordination also occurs through the meth­oxy O atom. The Ni^II^ atoms are linked by a μ_2_-oxide atom and one end of a μ_2_-azide ligand, forming an Ni_2_ON core. The coordination geometry for the Ni^II^ atom coordinated by the tridentate ligand is completed by the meth­oxy O atom derived from the penta­dentate ligand, with the resulting N_3_O_3_ donor set defining a *fac* octa­hedron. The second Ni^II^ atom has its *cis*-octa­hedral N_4_O_2_ coordination geometry completed by the imine N and amine N atoms of the penta­dentate Schiff base ligand, a terminally coordinated azide N and a methanol O atom. The arrangement is stabilized by an intra­molecular hydrogen bond between the methanol H and the oxide O atom. Linear supra­molecular chains along the *a* axis are formed in the crystal packing whereby two amine H atoms from different amine atoms hydrogen bond to the terminal N atom of the monodentate azide ligand.

## Related literature

For background to azido derivatives of tridentate Schiff base Ni^II^ complexes, see: Ribas *et al.* (1999 ▶); Koner *et al.* (2009 ▶); Biswas *et al.* (2011 ▶). For a related structure, see: Ghaemi *et al.* (2012 ▶).
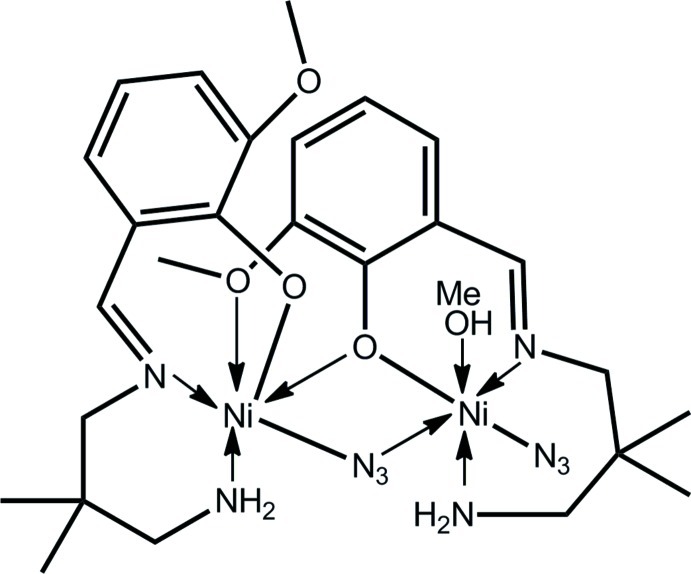


## Experimental

### 

#### Crystal data

[Ni_2_(C_13_H_19_N_2_O_2_)_2_(N_3_)_2_(CH_4_O)]
*M* *_r_* = 704.13
Monoclinic, 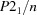

*a* = 8.0907 (2) Å
*b* = 18.5230 (4) Å
*c* = 21.1162 (4) Å
β = 96.674 (2)°
*V* = 3143.11 (12) Å^3^
*Z* = 4
Mo *K*α radiation
μ = 1.25 mm^−1^
*T* = 100 K
0.24 × 0.18 × 0.18 mm

#### Data collection

Agilent SuperNova Dual diffractometer with an Atlas detector
Absorption correction: multi-scan (*CrysAlis PRO*; Agilent, 2010 ▶) *T*_min_ = 0.753, *T*_max_ = 0.806
21789 measured reflections
7266 independent reflections
6115 reflections with *I* > 2σ(*I*)
*R*_int_ = 0.031

#### Refinement

*R*[*F* ^2^ > 2σ(*F* ^2^)] = 0.031
*wR*(*F* ^2^) = 0.076
*S* = 1.01
7266 reflections
417 parameters
5 restraints
H atoms treated by a mixture of independent and constrained refinement
Δρ_max_ = 0.49 e Å^−3^
Δρ_min_ = −0.44 e Å^−3^

### 

Data collection: *CrysAlis PRO* (Agilent, 2010 ▶); cell refinement: *CrysAlis PRO*; data reduction: *CrysAlis PRO*; program(s) used to solve structure: *SHELXS97* (Sheldrick, 2008 ▶); program(s) used to refine structure: *SHELXL97* (Sheldrick, 2008 ▶); molecular graphics: *ORTEP-3 for Windows* (Farrugia, 1997 ▶) and *DIAMOND* (Brandenburg, 2006 ▶); software used to prepare material for publication: *publCIF* (Westrip, 2010 ▶).

## Supplementary Material

Crystal structure: contains datablock(s) global, I. DOI: 10.1107/S1600536812029662/lh5496sup1.cif

Structure factors: contains datablock(s) I. DOI: 10.1107/S1600536812029662/lh5496Isup2.hkl

Additional supplementary materials:  crystallographic information; 3D view; checkCIF report

## Figures and Tables

**Table 1:** Selected bond lengths (Å)

Ni1—O2	2.0155 (14)
Ni1—O3	2.2589 (13)
Ni1—O4	2.0201 (13)
Ni1—N1	2.0166 (16)
Ni1—N2	2.0621 (17)
Ni1—N5	2.0862 (16)
Ni2—O4	2.0451 (13)
Ni2—O5	2.1364 (14)
Ni2—N3	2.0478 (16)
Ni2—N5	2.1505 (16)
Ni2—N4	2.0797 (16)
Ni2—N8	2.0715 (17)

**Table 2:** Hydrogen-bond geometry (Å, °)

*D*—H⋯*A*	*D*—H	H⋯*A*	*D*⋯*A*	*D*—H⋯*A*
O5—H5⋯O2	0.83 (1)	1.80 (1)	2.604 (2)	161 (3)
N2—H22⋯N10^i^	0.88 (1)	2.32 (2)	3.121 (2)	153 (2)
N4—H42⋯N10^i^	0.87 (1)	2.19 (1)	3.040 (2)	165 (2)

Symmetry code: (i) 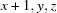.

**Fig. 1.**
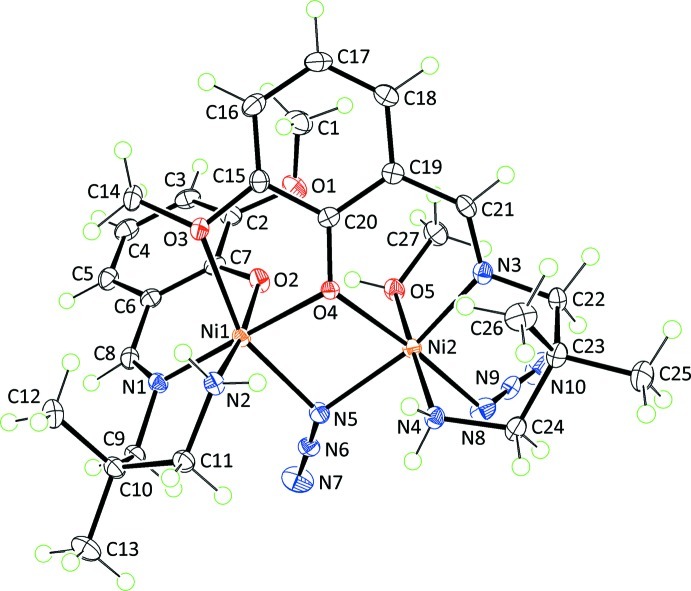
The molecular structure of (I) showing the atom-labelling scheme and displacement ellipsoids at the 50% probability level.

**Fig. 2.**
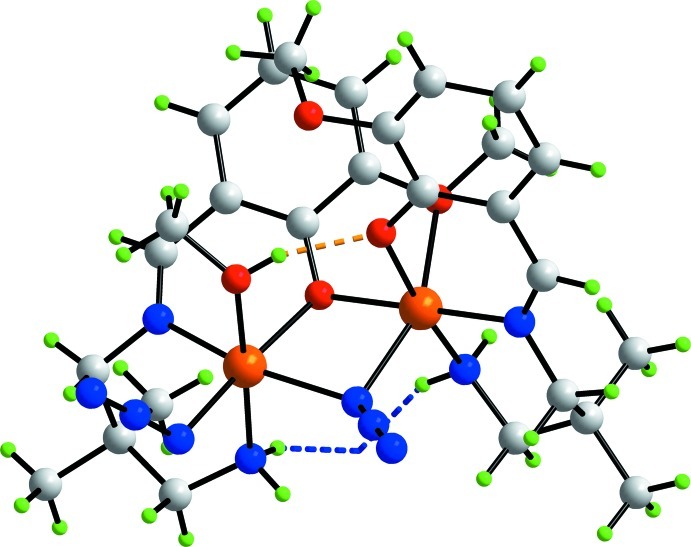
A view of the hydrogen bonding in (I) showing the formation of seven- and eight-membered synthons. The O—H···O and N—H···N hydrogen bonds are shown as orange and blue dashed lines, respectively.

**Fig. 3.**
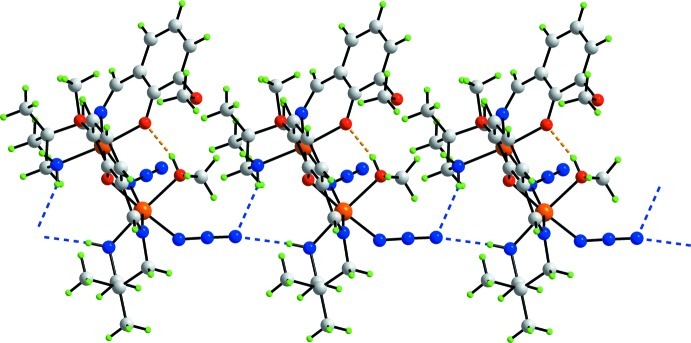
A view of the supramolecular chain in (I) propagated along the a axis. The O—H···O and N—H···N hydrogen bonds are shown as orange and blue dashed lines, respectively.

## supplementary crystallographic information

### Comment

The design and magnetism of polynuclear complexes containing paramagnetic centres connected through pseudo-halide bridges have attracted significant recent interest owing to their importance in understanding the basics of magnetic interactions and magneto-structural correlations with relevance to condensed matter physics, materials chemistry and coordination chemistry. Amongst these materials investigated, and relevant to the present report describing the crystal structure determination of the title complex (I), are azido derivatives of tridentate Schiff base Ni^II^ structures (Ribas *et al.*, 1999; Koner *et al.*, 2009; Biswas *et al.*, 2011). Recently, we described the structure of a centrosymmetric Cu^II^ complex which featured asymmetrically bridging azido ligands and a tridentate mode of coordination of the Schiff base ligand (Ghaemi *et al.*, 2012). Herein, a related binuclear Ni^II^ complex (I) is described.

In the binuclear complex (I), Fig. 1, the Ni^II^ atoms are bridged by a µ_2_-oxido atom and one end of a µ_2_-azido ligand to generate a Ni_2_ON core. The coordination geometry for the Ni1 atom is completed by a methoxy-O atom derived from the same ligand that provides the µ_2_-oxido bridge and the oxido-O, imine-O and amine-N donor atoms derived from a tridentate uninegative Schiff base ligand. The coordination geometry about the Ni2 atom is completed by the imine-N and amine-N atoms of the original Schiff base ligand, indicating that this is pentadentate, a terminally coordinate azido-N and a methanol-O atom. The N_3_O_3_ donor set for the Ni1 atom defines a *fac*-octahedron, whereas the N_4_O_2_ donor set for the Ni2 atom defines a *cis*-octahedron. Table 1 collects the Ni—*L* bond lengths and shows that the µ_2_-oxido bridge is symmetric but some asymmetry is present in the µ_2_-azido bridge. The longest Ni—O bond lengths for each Ni atom involves methoxy-O (Ni1) and methanol-O (Ni2). As expected, the Ni—N(terminal azide) bond is shorter than the Ni—N bridging distances. The Ni—*N*(imine) bond lengths are the shorter of the Ni—N bond lengths for the two environments.

Hydrogen bonding occurs in the structure, Table 1. The methanol-H forms an intramolecular hydrogen bond to the oxido-O2 atom to close six-membered {···HONiONiO} and {···HONiNNiO} synthons, Fig. 2. Two of the amine-H atoms form hydrogen bonds to the terminal-N10 atom of the monodentate azido ligand to form eight-membered {···HNNiONiNH···N} and {···HNNiNNiNH···N} synthons, Fig. 2, and a linear supramolecular chain along the *a* axis, Fig. 3.

### Experimental

To prepare this complex, a methanolic solution (40 ml) of 2,2'-dimethylpropylenediamine (1 mmol, 0.102 g) was first mixed with 2-hydroxy-3-methoxybenzaldehyde (2 mmol, 0.304 g) under stirring to prepare the desired Schiff-base *in situ*. Stirring was continued for 30 min. Then, Cu(NO_3_)_2_.3H_2_O (0.120 g, 0.5 mmol) and Ni(NO_3_)2.6H_2_O (0.145 g, 0.5 mmol) dissolved in methanol (20 ml) was added to the solution and the resulting mixture was stirred for about 10 min. Finally, an aqueous solution of NaN_3_ (2 ml, 8 mmol, 0.52 g) was added drop-wise to the resulting mixture with continuous stirring, and the solution was filtered. Dark-green crystals were formed within few days from the filtered solution. Analysis confirmed the formation of a di-nickel(II) complex rather than the anticipated hetero-metallic complex, as confirmed by X-ray crystallography. Anal. Calc. for C_27_H_42_N_10_Ni_2_O_5_: C, 46.06; H, 6.01; N, 19.89. Found: C, 45.93; H, 5.85; N, 19.76%. IR (KBr) [cm^-1^]: ν_as_(N_3_) 2047, 2068 *vs*, ν(C═N) 1620 s, ν(C═C) 1540 s, ν(C—O) 1224 m, ν(O—H) 3340 b. Yield: 56%, *M*.pt: 544–548 K.

### Refinement

Carbon-bound H-atoms were placed in calculated positions [C—H = 0.95–0.99 Å, *U*_iso_(H) = 1.2–1.5*U*_eq_(C)] and were included in the refinement in the riding model approximation. The hydroxyl-H and amine-H H-atoms were located from a difference map and refined with O—H = 0.84±0.01 Å and N—H = 0.88±0.01 Å, respectively, and with *U_iso_*(H) = 1.5*U_eq_*(O) and 1.2*U_eq_*(N).

### Crystal data

**Table:**

[Ni_2_(C_13_H_19_N_2_O_2_)_2_(N_3_)_2_(CH_4_O)]	*F*(000) = 1480
*M**_r_* = 704.13	*D*_x_ = 1.488 Mg m^−^^3^
Monoclinic, *P*2_1_/*n*	Mo *K*α radiation, λ = 0.71073 Å
Hall symbol: -P 2yn	Cell parameters from 9647 reflections
*a* = 8.0907 (2) Å	θ = 2.4–27.5°
*b* = 18.5230 (4) Å	µ = 1.25 mm^−^^1^
*c* = 21.1162 (4) Å	*T* = 100 K
β = 96.674 (2)°	Prism, green
*V* = 3143.11 (12) Å^3^	0.24 × 0.18 × 0.18 mm
*Z* = 4	

### Data collection

**Table:**

Agilent SuperNova Dual diffractometer with an Atlas detector	7266 independent reflections
Radiation source: SuperNova (Mo) X-ray Source	6115 reflections with *I* > 2σ(*I*)
Mirror monochromator	*R*_int_ = 0.031
Detector resolution: 10.4041 pixels mm^-1^	θ_max_ = 27.6°, θ_min_ = 2.4°
ω scan	*h* = −10→9
Absorption correction: multi-scan (*CrysAlis PRO*; Agilent, 2010)	*k* = −24→23
*T*_min_ = 0.753, *T*_max_ = 0.806	*l* = −20→27
21789 measured reflections	

### Refinement

**Table:**

Refinement on *F*^2^	Primary atom site location: structure-invariant direct methods
Least-squares matrix: full	Secondary atom site location: difference Fourier map
*R*[*F*^2^ > 2σ(*F*^2^)] = 0.031	Hydrogen site location: inferred from neighbouring sites
*wR*(*F*^2^) = 0.076	H atoms treated by a mixture of independent and constrained refinement
*S* = 1.01	*w* = 1/[σ^2^(*F*_o_^2^) + (0.0296*P*)^2^ + 2.1117*P*] where *P* = (*F*_o_^2^ + 2*F*_c_^2^)/3
7266 reflections	(Δ/σ)_max_ = 0.001
417 parameters	Δρ_max_ = 0.49 e Å^−^^3^
5 restraints	Δρ_min_ = −0.44 e Å^−^^3^

### Fractional atomic coordinates and isotropic or equivalent isotropic displacement parameters (Å^2^)

**Table:**

	*x*	*y*	*z*	*U*_iso_*/*U*_eq_	
Ni1	0.76377 (3)	0.497301 (13)	0.206798 (11)	0.01246 (7)	
Ni2	0.60168 (3)	0.568768 (13)	0.320859 (11)	0.01225 (7)	
O1	0.30566 (18)	0.34321 (8)	0.21466 (7)	0.0224 (3)	
O2	0.55396 (17)	0.43769 (7)	0.19832 (6)	0.0175 (3)	
O3	0.88920 (17)	0.39094 (7)	0.23383 (6)	0.0169 (3)	
O4	0.77159 (16)	0.49200 (7)	0.30265 (6)	0.0123 (3)	
O5	0.40544 (18)	0.49563 (7)	0.28883 (7)	0.0174 (3)	
H5	0.438 (3)	0.4697 (12)	0.2606 (9)	0.038 (8)*	
N1	0.7467 (2)	0.50350 (8)	0.11091 (7)	0.0141 (3)	
N2	0.9982 (2)	0.54253 (9)	0.21894 (8)	0.0158 (3)	
H21	1.072 (2)	0.5079 (9)	0.2186 (11)	0.020 (6)*	
H22	1.007 (3)	0.5592 (12)	0.2581 (6)	0.032 (7)*	
N3	0.63088 (19)	0.53036 (9)	0.41241 (7)	0.0146 (3)	
N4	0.7772 (2)	0.64761 (9)	0.34998 (8)	0.0146 (3)	
H41	0.778 (3)	0.6781 (9)	0.3171 (7)	0.012 (5)*	
H42	0.8797 (15)	0.6324 (13)	0.3523 (12)	0.034 (7)*	
N5	0.6386 (2)	0.59256 (9)	0.22382 (7)	0.0152 (3)	
N6	0.5399 (2)	0.61611 (9)	0.18170 (8)	0.0157 (3)	
N7	0.4510 (2)	0.63934 (10)	0.14030 (9)	0.0279 (4)	
N8	0.4217 (2)	0.64681 (9)	0.32858 (8)	0.0197 (4)	
N9	0.2826 (2)	0.63245 (9)	0.33620 (8)	0.0170 (3)	
N10	0.1456 (2)	0.62014 (11)	0.34411 (10)	0.0305 (5)	
C1	0.3810 (3)	0.28869 (12)	0.25635 (11)	0.0339 (6)	
H1A	0.3312	0.2894	0.2965	0.051*	
H1B	0.5008	0.2979	0.2650	0.051*	
H1C	0.3629	0.2413	0.2361	0.051*	
C2	0.3687 (2)	0.34659 (11)	0.15632 (9)	0.0177 (4)	
C3	0.3041 (3)	0.30317 (11)	0.10674 (10)	0.0196 (4)	
H3	0.2214	0.2685	0.1134	0.024*	
C4	0.3589 (3)	0.30964 (11)	0.04667 (10)	0.0213 (4)	
H4	0.3148	0.2792	0.0126	0.026*	
C5	0.4770 (3)	0.36038 (11)	0.03738 (9)	0.0188 (4)	
H5A	0.5127	0.3655	−0.0037	0.023*	
C6	0.5466 (2)	0.40518 (10)	0.08773 (9)	0.0152 (4)	
C7	0.4949 (2)	0.39842 (10)	0.14951 (9)	0.0151 (4)	
C8	0.6576 (2)	0.46182 (10)	0.07183 (9)	0.0158 (4)	
H8	0.6657	0.4691	0.0278	0.019*	
C9	0.8245 (2)	0.56541 (10)	0.08267 (9)	0.0154 (4)	
H9A	0.7621	0.6095	0.0918	0.018*	
H9B	0.8118	0.5590	0.0358	0.018*	
C10	1.0093 (2)	0.57828 (10)	0.10513 (9)	0.0164 (4)	
C11	1.0368 (2)	0.60104 (10)	0.17535 (9)	0.0163 (4)	
H11A	1.1542	0.6159	0.1862	0.020*	
H11B	0.9656	0.6433	0.1817	0.020*	
C12	1.1134 (3)	0.51171 (11)	0.09389 (10)	0.0220 (4)	
H12A	1.2305	0.5213	0.1089	0.033*	
H12B	1.1021	0.5005	0.0482	0.033*	
H12C	1.0743	0.4706	0.1173	0.033*	
C13	1.0618 (3)	0.64185 (12)	0.06528 (10)	0.0232 (5)	
H13A	1.1797	0.6526	0.0776	0.035*	
H13B	0.9948	0.6844	0.0729	0.035*	
H13C	1.0442	0.6292	0.0199	0.035*	
C14	0.9007 (3)	0.33467 (11)	0.18754 (9)	0.0204 (4)	
H14A	0.9590	0.2930	0.2082	0.031*	
H14B	0.9625	0.3525	0.1534	0.031*	
H14C	0.7886	0.3202	0.1694	0.031*	
C15	0.8407 (2)	0.36947 (10)	0.29201 (9)	0.0151 (4)	
C16	0.8521 (3)	0.29996 (11)	0.31517 (10)	0.0200 (4)	
H16	0.8952	0.2626	0.2909	0.024*	
C17	0.7998 (3)	0.28455 (11)	0.37473 (10)	0.0206 (4)	
H17	0.8056	0.2365	0.3906	0.025*	
C18	0.7403 (2)	0.33867 (11)	0.40992 (9)	0.0180 (4)	
H18	0.7066	0.3278	0.4505	0.022*	
C19	0.7280 (2)	0.41017 (10)	0.38728 (9)	0.0146 (4)	
C20	0.7773 (2)	0.42644 (10)	0.32705 (9)	0.0129 (4)	
C21	0.6803 (2)	0.46681 (10)	0.42907 (9)	0.0149 (4)	
H21A	0.6865	0.4556	0.4732	0.018*	
C22	0.6171 (3)	0.58335 (11)	0.46303 (9)	0.0181 (4)	
H22A	0.6166	0.5579	0.5042	0.022*	
H22B	0.5104	0.6096	0.4542	0.022*	
C23	0.7622 (3)	0.63805 (11)	0.46827 (9)	0.0187 (4)	
C24	0.7553 (2)	0.68694 (10)	0.40948 (9)	0.0169 (4)	
H24A	0.6466	0.7121	0.4039	0.020*	
H24B	0.8433	0.7241	0.4170	0.020*	
C25	0.7405 (3)	0.68711 (12)	0.52552 (10)	0.0301 (5)	
H25A	0.7442	0.6578	0.5643	0.045*	
H25B	0.6330	0.7120	0.5182	0.045*	
H25C	0.8303	0.7228	0.5306	0.045*	
C26	0.9292 (3)	0.59914 (12)	0.47949 (10)	0.0244 (5)	
H26A	0.9307	0.5678	0.5169	0.037*	
H26B	1.0190	0.6347	0.4868	0.037*	
H26C	0.9452	0.5699	0.4420	0.037*	
C27	0.3146 (3)	0.45380 (12)	0.33012 (10)	0.0216 (4)	
H27A	0.2309	0.4246	0.3045	0.032*	
H27B	0.2593	0.4862	0.3577	0.032*	
H27C	0.3914	0.4220	0.3564	0.032*	

### Atomic displacement parameters (Å^2^)

**Table:**

	*U*^11^	*U*^22^	*U*^33^	*U*^12^	*U*^13^	*U*^23^
Ni1	0.01189 (13)	0.01354 (12)	0.01216 (12)	−0.00137 (9)	0.00225 (9)	−0.00016 (9)
Ni2	0.01019 (12)	0.01284 (12)	0.01395 (12)	0.00028 (9)	0.00238 (9)	0.00016 (9)
O1	0.0190 (8)	0.0268 (8)	0.0220 (7)	−0.0050 (6)	0.0044 (6)	0.0041 (6)
O2	0.0159 (7)	0.0219 (7)	0.0147 (6)	−0.0061 (6)	0.0026 (5)	−0.0028 (6)
O3	0.0204 (7)	0.0150 (7)	0.0162 (7)	0.0005 (6)	0.0061 (5)	−0.0026 (5)
O4	0.0124 (6)	0.0121 (6)	0.0128 (6)	0.0007 (5)	0.0029 (5)	0.0007 (5)
O5	0.0150 (7)	0.0192 (7)	0.0187 (7)	−0.0035 (6)	0.0053 (6)	−0.0009 (6)
N1	0.0121 (8)	0.0156 (8)	0.0150 (8)	−0.0007 (6)	0.0030 (6)	0.0012 (6)
N2	0.0155 (9)	0.0180 (8)	0.0144 (8)	−0.0009 (7)	0.0033 (7)	−0.0001 (7)
N3	0.0108 (8)	0.0171 (8)	0.0164 (8)	−0.0018 (6)	0.0040 (6)	−0.0016 (7)
N4	0.0117 (8)	0.0161 (8)	0.0165 (8)	−0.0001 (7)	0.0038 (6)	−0.0013 (7)
N5	0.0138 (8)	0.0174 (8)	0.0145 (8)	0.0021 (7)	0.0019 (6)	0.0017 (6)
N6	0.0129 (8)	0.0142 (8)	0.0207 (8)	−0.0034 (7)	0.0041 (7)	0.0006 (7)
N7	0.0202 (10)	0.0327 (10)	0.0290 (10)	0.0018 (8)	−0.0049 (8)	0.0104 (9)
N8	0.0109 (8)	0.0179 (8)	0.0304 (9)	0.0021 (7)	0.0029 (7)	−0.0006 (7)
N9	0.0153 (9)	0.0165 (8)	0.0187 (8)	0.0028 (7)	0.0001 (7)	−0.0052 (7)
N10	0.0156 (9)	0.0310 (11)	0.0468 (12)	−0.0016 (8)	0.0107 (8)	−0.0135 (9)
C1	0.0513 (16)	0.0271 (12)	0.0223 (11)	0.0001 (11)	0.0005 (11)	0.0040 (10)
C2	0.0154 (10)	0.0186 (10)	0.0188 (10)	0.0019 (8)	0.0007 (8)	0.0023 (8)
C3	0.0158 (10)	0.0141 (9)	0.0281 (11)	−0.0027 (8)	−0.0016 (8)	0.0001 (8)
C4	0.0233 (11)	0.0167 (10)	0.0223 (10)	−0.0013 (8)	−0.0034 (9)	−0.0044 (8)
C5	0.0212 (11)	0.0198 (10)	0.0146 (9)	0.0032 (8)	−0.0006 (8)	−0.0024 (8)
C6	0.0142 (10)	0.0140 (9)	0.0169 (9)	0.0009 (8)	−0.0005 (7)	−0.0003 (8)
C7	0.0136 (9)	0.0144 (9)	0.0164 (9)	0.0025 (7)	−0.0020 (7)	0.0003 (8)
C8	0.0158 (10)	0.0186 (10)	0.0129 (9)	0.0041 (8)	0.0017 (7)	−0.0004 (8)
C9	0.0160 (10)	0.0161 (9)	0.0144 (9)	−0.0006 (8)	0.0033 (7)	0.0024 (8)
C10	0.0157 (10)	0.0182 (10)	0.0160 (9)	−0.0009 (8)	0.0046 (7)	0.0025 (8)
C11	0.0158 (10)	0.0166 (9)	0.0169 (9)	−0.0019 (8)	0.0036 (7)	0.0003 (8)
C12	0.0202 (11)	0.0271 (11)	0.0199 (10)	0.0021 (9)	0.0067 (8)	−0.0019 (9)
C13	0.0176 (10)	0.0275 (11)	0.0246 (11)	−0.0047 (9)	0.0030 (8)	0.0080 (9)
C14	0.0242 (11)	0.0195 (10)	0.0186 (10)	0.0016 (9)	0.0076 (8)	−0.0051 (8)
C15	0.0130 (9)	0.0174 (9)	0.0151 (9)	−0.0003 (8)	0.0025 (7)	−0.0012 (8)
C16	0.0233 (11)	0.0142 (9)	0.0223 (10)	0.0026 (8)	0.0020 (8)	−0.0028 (8)
C17	0.0250 (11)	0.0138 (9)	0.0225 (10)	−0.0001 (8)	−0.0001 (8)	0.0039 (8)
C18	0.0183 (10)	0.0195 (10)	0.0157 (9)	−0.0012 (8)	0.0002 (8)	0.0035 (8)
C19	0.0121 (9)	0.0157 (9)	0.0156 (9)	−0.0006 (7)	0.0003 (7)	0.0004 (8)
C20	0.0095 (9)	0.0130 (9)	0.0157 (9)	−0.0013 (7)	−0.0007 (7)	−0.0007 (7)
C21	0.0133 (9)	0.0192 (10)	0.0125 (9)	−0.0031 (8)	0.0031 (7)	0.0014 (8)
C22	0.0217 (11)	0.0192 (10)	0.0147 (9)	0.0026 (8)	0.0075 (8)	−0.0008 (8)
C23	0.0222 (11)	0.0178 (10)	0.0163 (9)	0.0005 (8)	0.0030 (8)	−0.0038 (8)
C24	0.0162 (10)	0.0152 (9)	0.0196 (10)	0.0000 (8)	0.0038 (8)	−0.0043 (8)
C25	0.0452 (15)	0.0250 (11)	0.0212 (11)	−0.0049 (11)	0.0093 (10)	−0.0086 (9)
C26	0.0217 (11)	0.0240 (11)	0.0254 (11)	−0.0002 (9)	−0.0055 (9)	0.0009 (9)
C27	0.0165 (10)	0.0256 (11)	0.0233 (10)	−0.0035 (8)	0.0046 (8)	0.0013 (9)

### Geometric parameters (Å, º)

**Table:**

Ni1—O2	2.0155 (14)	C8—H8	0.9500
Ni1—O3	2.2589 (13)	C9—C10	1.534 (3)
Ni1—O4	2.0201 (13)	C9—H9A	0.9900
Ni1—N1	2.0166 (16)	C9—H9B	0.9900
Ni1—N2	2.0621 (17)	C10—C12	1.527 (3)
Ni1—N5	2.0862 (16)	C10—C11	1.533 (3)
Ni2—O4	2.0451 (13)	C10—C13	1.535 (3)
Ni2—O5	2.1364 (14)	C11—H11A	0.9900
Ni2—N3	2.0478 (16)	C11—H11B	0.9900
Ni2—N5	2.1505 (16)	C12—H12A	0.9800
Ni2—N4	2.0797 (16)	C12—H12B	0.9800
Ni2—N8	2.0715 (17)	C12—H12C	0.9800
O1—C2	1.388 (2)	C13—H13A	0.9800
O1—C1	1.429 (3)	C13—H13B	0.9800
O2—C7	1.306 (2)	C13—H13C	0.9800
O3—C15	1.390 (2)	C14—H14A	0.9800
O3—C14	1.439 (2)	C14—H14B	0.9800
O4—C20	1.318 (2)	C14—H14C	0.9800
O5—C27	1.432 (2)	C15—C16	1.377 (3)
O5—H5	0.832 (10)	C15—C20	1.419 (3)
N1—C8	1.288 (2)	C16—C17	1.402 (3)
N1—C9	1.468 (2)	C16—H16	0.9500
N2—C11	1.478 (2)	C17—C18	1.368 (3)
N2—H21	0.879 (10)	C17—H17	0.9500
N2—H22	0.877 (10)	C18—C19	1.408 (3)
N3—C21	1.279 (2)	C18—H18	0.9500
N3—C22	1.465 (2)	C19—C20	1.409 (3)
N4—C24	1.481 (2)	C19—C21	1.452 (3)
N4—H41	0.895 (9)	C21—H21A	0.9500
N4—H42	0.872 (10)	C22—C23	1.545 (3)
N5—N6	1.206 (2)	C22—H22A	0.9900
N6—N7	1.150 (2)	C22—H22B	0.9900
N8—N9	1.186 (2)	C23—C26	1.525 (3)
N9—N10	1.162 (2)	C23—C24	1.532 (3)
C1—H1A	0.9800	C23—C25	1.539 (3)
C1—H1B	0.9800	C24—H24A	0.9900
C1—H1C	0.9800	C24—H24B	0.9900
C2—C3	1.375 (3)	C25—H25A	0.9800
C2—C7	1.421 (3)	C25—H25B	0.9800
C3—C4	1.397 (3)	C25—H25C	0.9800
C3—H3	0.9500	C26—H26A	0.9800
C4—C5	1.370 (3)	C26—H26B	0.9800
C4—H4	0.9500	C26—H26C	0.9800
C5—C6	1.413 (3)	C27—H27A	0.9800
C5—H5A	0.9500	C27—H27B	0.9800
C6—C7	1.421 (3)	C27—H27C	0.9800
C6—C8	1.445 (3)		
			
N1—Ni1—O2	89.03 (6)	N1—C9—H9A	108.2
N1—Ni1—O4	177.81 (6)	C10—C9—H9A	108.2
O2—Ni1—O4	89.45 (5)	N1—C9—H9B	108.2
N1—Ni1—N2	93.24 (7)	C10—C9—H9B	108.2
O2—Ni1—N2	170.63 (6)	H9A—C9—H9B	107.3
O4—Ni1—N2	88.51 (6)	C12—C10—C9	111.21 (16)
N1—Ni1—N5	98.44 (6)	C12—C10—C11	110.60 (16)
O2—Ni1—N5	93.33 (6)	C9—C10—C11	111.61 (16)
O4—Ni1—N5	80.08 (6)	C12—C10—C13	109.99 (17)
N2—Ni1—N5	95.32 (7)	C9—C10—C13	105.73 (15)
N1—Ni1—O3	106.25 (6)	C11—C10—C13	107.52 (16)
O2—Ni1—O3	83.89 (5)	N2—C11—C10	112.55 (15)
O4—Ni1—O3	75.14 (5)	N2—C11—H11A	109.1
N2—Ni1—O3	86.75 (6)	C10—C11—H11A	109.1
N5—Ni1—O3	155.08 (6)	N2—C11—H11B	109.1
O4—Ni2—N3	85.96 (6)	C10—C11—H11B	109.1
O4—Ni2—N8	173.69 (6)	H11A—C11—H11B	107.8
N3—Ni2—N8	99.97 (7)	C10—C12—H12A	109.5
O4—Ni2—N4	95.33 (6)	C10—C12—H12B	109.5
N3—Ni2—N4	87.99 (6)	H12A—C12—H12B	109.5
N8—Ni2—N4	87.06 (7)	C10—C12—H12C	109.5
O4—Ni2—O5	89.46 (5)	H12A—C12—H12C	109.5
N3—Ni2—O5	94.50 (6)	H12B—C12—H12C	109.5
N8—Ni2—O5	87.95 (6)	C10—C13—H13A	109.5
N4—Ni2—O5	174.74 (6)	C10—C13—H13B	109.5
O4—Ni2—N5	78.02 (6)	H13A—C13—H13B	109.5
N3—Ni2—N5	163.17 (6)	C10—C13—H13C	109.5
N8—Ni2—N5	96.25 (7)	H13A—C13—H13C	109.5
N4—Ni2—N5	88.39 (6)	H13B—C13—H13C	109.5
O5—Ni2—N5	90.50 (6)	O3—C14—H14A	109.5
C2—O1—C1	113.83 (17)	O3—C14—H14B	109.5
C7—O2—Ni1	127.05 (13)	H14A—C14—H14B	109.5
C15—O3—C14	116.12 (15)	O3—C14—H14C	109.5
C15—O3—Ni1	107.95 (11)	H14A—C14—H14C	109.5
C14—O3—Ni1	121.51 (11)	H14B—C14—H14C	109.5
C20—O4—Ni1	115.60 (11)	C16—C15—O3	124.56 (18)
C20—O4—Ni2	124.11 (12)	C16—C15—C20	121.58 (18)
Ni1—O4—Ni2	102.19 (5)	O3—C15—C20	113.86 (16)
C27—O5—Ni2	124.44 (12)	C15—C16—C17	119.63 (19)
C27—O5—H5	110.5 (19)	C15—C16—H16	120.2
Ni2—O5—H5	108.1 (19)	C17—C16—H16	120.2
C8—N1—C9	116.27 (16)	C18—C17—C16	120.00 (18)
C8—N1—Ni1	125.26 (14)	C18—C17—H17	120.0
C9—N1—Ni1	118.02 (12)	C16—C17—H17	120.0
C11—N2—Ni1	118.56 (12)	C17—C18—C19	121.35 (19)
C11—N2—H21	109.6 (16)	C17—C18—H18	119.3
Ni1—N2—H21	108.7 (15)	C19—C18—H18	119.3
C11—N2—H22	109.2 (16)	C18—C19—C20	119.45 (18)
Ni1—N2—H22	103.3 (17)	C18—C19—C21	119.13 (18)
H21—N2—H22	107 (2)	C20—C19—C21	121.14 (17)
C21—N3—C22	117.64 (16)	O4—C20—C19	123.33 (17)
C21—N3—Ni2	125.26 (13)	O4—C20—C15	118.66 (17)
C22—N3—Ni2	116.48 (12)	C19—C20—C15	117.98 (17)
C24—N4—Ni2	116.81 (12)	N3—C21—C19	126.49 (17)
C24—N4—H41	111.1 (13)	N3—C21—H21A	116.8
Ni2—N4—H41	106.3 (13)	C19—C21—H21A	116.8
C24—N4—H42	108.6 (16)	N3—C22—C23	111.72 (16)
Ni2—N4—H42	113.7 (16)	N3—C22—H22A	109.3
H41—N4—H42	99 (2)	C23—C22—H22A	109.3
N6—N5—Ni1	118.16 (13)	N3—C22—H22B	109.3
N6—N5—Ni2	128.57 (14)	C23—C22—H22B	109.3
Ni1—N5—Ni2	96.60 (6)	H22A—C22—H22B	107.9
N7—N6—N5	177.3 (2)	C26—C23—C24	110.71 (18)
N9—N8—Ni2	122.77 (14)	C26—C23—C25	109.70 (17)
N10—N9—N8	178.3 (2)	C24—C23—C25	106.90 (16)
O1—C1—H1A	109.5	C26—C23—C22	110.68 (17)
O1—C1—H1B	109.5	C24—C23—C22	111.93 (16)
H1A—C1—H1B	109.5	C25—C23—C22	106.75 (17)
O1—C1—H1C	109.5	N4—C24—C23	113.58 (16)
H1A—C1—H1C	109.5	N4—C24—H24A	108.8
H1B—C1—H1C	109.5	C23—C24—H24A	108.8
C3—C2—O1	120.20 (18)	N4—C24—H24B	108.8
C3—C2—C7	122.17 (19)	C23—C24—H24B	108.8
O1—C2—C7	117.56 (17)	H24A—C24—H24B	107.7
C2—C3—C4	120.57 (19)	C23—C25—H25A	109.5
C2—C3—H3	119.7	C23—C25—H25B	109.5
C4—C3—H3	119.7	H25A—C25—H25B	109.5
C5—C4—C3	119.29 (18)	C23—C25—H25C	109.5
C5—C4—H4	120.4	H25A—C25—H25C	109.5
C3—C4—H4	120.4	H25B—C25—H25C	109.5
C4—C5—C6	121.24 (19)	C23—C26—H26A	109.5
C4—C5—H5A	119.4	C23—C26—H26B	109.5
C6—C5—H5A	119.4	H26A—C26—H26B	109.5
C5—C6—C7	120.30 (18)	C23—C26—H26C	109.5
C5—C6—C8	117.10 (18)	H26A—C26—H26C	109.5
C7—C6—C8	122.30 (17)	H26B—C26—H26C	109.5
O2—C7—C6	123.75 (18)	O5—C27—H27A	109.5
O2—C7—C2	119.85 (18)	O5—C27—H27B	109.5
C6—C7—C2	116.38 (17)	H27A—C27—H27B	109.5
N1—C8—C6	127.07 (18)	O5—C27—H27C	109.5
N1—C8—H8	116.5	H27A—C27—H27C	109.5
C6—C8—H8	116.5	H27B—C27—H27C	109.5
N1—C9—C10	116.40 (15)		
			
N1—Ni1—O2—C7	24.42 (15)	O4—Ni2—N5—Ni1	12.98 (5)
O4—Ni1—O2—C7	−157.15 (15)	N3—Ni2—N5—Ni1	31.1 (2)
N5—Ni1—O2—C7	122.82 (15)	N8—Ni2—N5—Ni1	−164.35 (6)
O3—Ni1—O2—C7	−82.03 (15)	N4—Ni2—N5—Ni1	108.78 (7)
N1—Ni1—O3—C15	−154.40 (11)	O5—Ni2—N5—Ni1	−76.36 (6)
O2—Ni1—O3—C15	−67.20 (11)	N3—Ni2—N8—N9	−69.32 (17)
O4—Ni1—O3—C15	23.85 (11)	N4—Ni2—N8—N9	−156.76 (17)
N2—Ni1—O3—C15	113.17 (12)	O5—Ni2—N8—N9	24.90 (16)
N5—Ni1—O3—C15	17.54 (19)	N5—Ni2—N8—N9	115.18 (17)
N1—Ni1—O3—C14	−16.55 (15)	C1—O1—C2—C3	86.4 (2)
O2—Ni1—O3—C14	70.65 (14)	C1—O1—C2—C7	−96.6 (2)
O4—Ni1—O3—C14	161.70 (15)	O1—C2—C3—C4	175.66 (18)
N2—Ni1—O3—C14	−108.98 (14)	C7—C2—C3—C4	−1.3 (3)
N5—Ni1—O3—C14	155.38 (15)	C2—C3—C4—C5	−0.7 (3)
O2—Ni1—O4—C20	58.20 (13)	C3—C4—C5—C6	1.3 (3)
N2—Ni1—O4—C20	−112.66 (13)	C4—C5—C6—C7	0.0 (3)
N5—Ni1—O4—C20	151.67 (13)	C4—C5—C6—C8	−173.92 (18)
O3—Ni1—O4—C20	−25.63 (12)	Ni1—O2—C7—C6	−16.5 (3)
O2—Ni1—O4—Ni2	−79.51 (6)	Ni1—O2—C7—C2	165.10 (13)
N2—Ni1—O4—Ni2	109.64 (7)	C5—C6—C7—O2	179.77 (17)
N5—Ni1—O4—Ni2	13.97 (6)	C8—C6—C7—O2	−6.7 (3)
O3—Ni1—O4—Ni2	−163.34 (6)	C5—C6—C7—C2	−1.8 (3)
N3—Ni2—O4—C20	38.68 (13)	C8—C6—C7—C2	171.77 (17)
N4—Ni2—O4—C20	126.28 (13)	C3—C2—C7—O2	−179.03 (18)
O5—Ni2—O4—C20	−55.88 (13)	O1—C2—C7—O2	4.0 (3)
N5—Ni2—O4—C20	−146.51 (14)	C3—C2—C7—C6	2.5 (3)
N3—Ni2—O4—Ni1	171.54 (6)	O1—C2—C7—C6	−174.56 (17)
N4—Ni2—O4—Ni1	−100.85 (6)	C9—N1—C8—C6	−168.84 (18)
O5—Ni2—O4—Ni1	76.99 (6)	Ni1—N1—C8—C6	3.3 (3)
N5—Ni2—O4—Ni1	−13.64 (6)	C5—C6—C8—N1	−172.40 (19)
O4—Ni2—O5—C27	100.30 (15)	C7—C6—C8—N1	13.9 (3)
N3—Ni2—O5—C27	14.39 (16)	C8—N1—C9—C10	−132.96 (18)
N8—Ni2—O5—C27	−85.45 (15)	Ni1—N1—C9—C10	54.3 (2)
N5—Ni2—O5—C27	178.32 (15)	N1—C9—C10—C12	56.7 (2)
O2—Ni1—N1—C8	−17.53 (16)	N1—C9—C10—C11	−67.3 (2)
N2—Ni1—N1—C8	153.38 (16)	N1—C9—C10—C13	176.07 (17)
N5—Ni1—N1—C8	−110.75 (16)	Ni1—N2—C11—C10	−56.9 (2)
O3—Ni1—N1—C8	65.83 (17)	C12—C10—C11—N2	−57.1 (2)
O2—Ni1—N1—C9	154.47 (14)	C9—C10—C11—N2	67.3 (2)
N2—Ni1—N1—C9	−34.62 (14)	C13—C10—C11—N2	−177.17 (16)
N5—Ni1—N1—C9	61.25 (14)	C14—O3—C15—C16	20.5 (3)
O3—Ni1—N1—C9	−122.17 (13)	Ni1—O3—C15—C16	160.90 (16)
N1—Ni1—N2—C11	37.14 (15)	C14—O3—C15—C20	−159.81 (16)
O4—Ni1—N2—C11	−141.55 (14)	Ni1—O3—C15—C20	−19.40 (18)
N5—Ni1—N2—C11	−61.65 (14)	O3—C15—C16—C17	179.80 (18)
O3—Ni1—N2—C11	143.25 (14)	C20—C15—C16—C17	0.1 (3)
O4—Ni2—N3—C21	−24.19 (16)	C15—C16—C17—C18	−1.0 (3)
N8—Ni2—N3—C21	153.66 (16)	C16—C17—C18—C19	0.9 (3)
N4—Ni2—N3—C21	−119.68 (17)	C17—C18—C19—C20	0.3 (3)
O5—Ni2—N3—C21	64.95 (16)	C17—C18—C19—C21	−173.75 (18)
N5—Ni2—N3—C21	−42.0 (3)	Ni1—O4—C20—C19	−157.92 (14)
O4—Ni2—N3—C22	146.46 (13)	Ni2—O4—C20—C19	−30.5 (2)
N8—Ni2—N3—C22	−35.69 (14)	Ni1—O4—C20—C15	24.0 (2)
N4—Ni2—N3—C22	50.97 (14)	Ni2—O4—C20—C15	151.43 (13)
O5—Ni2—N3—C22	−124.40 (13)	C18—C19—C20—O4	−179.20 (17)
N5—Ni2—N3—C22	128.7 (2)	C21—C19—C20—O4	−5.3 (3)
O4—Ni2—N4—C24	−133.28 (13)	C18—C19—C20—C15	−1.1 (3)
N3—Ni2—N4—C24	−47.52 (13)	C21—C19—C20—C15	172.73 (17)
N8—Ni2—N4—C24	52.57 (13)	C16—C15—C20—O4	179.12 (17)
N5—Ni2—N4—C24	148.92 (13)	O3—C15—C20—O4	−0.6 (2)
N1—Ni1—N5—N6	24.62 (16)	C16—C15—C20—C19	1.0 (3)
O2—Ni1—N5—N6	−64.89 (15)	O3—C15—C20—C19	−178.74 (16)
O4—Ni1—N5—N6	−153.75 (15)	C22—N3—C21—C19	−169.38 (18)
N2—Ni1—N5—N6	118.71 (15)	Ni2—N3—C21—C19	1.2 (3)
O3—Ni1—N5—N6	−147.55 (14)	C18—C19—C21—N3	−164.60 (19)
N1—Ni1—N5—Ni2	165.32 (6)	C20—C19—C21—N3	21.5 (3)
O2—Ni1—N5—Ni2	75.80 (6)	C21—N3—C22—C23	104.2 (2)
O4—Ni1—N5—Ni2	−13.05 (6)	Ni2—N3—C22—C23	−67.23 (18)
N2—Ni1—N5—Ni2	−100.60 (7)	N3—C22—C23—C26	−56.9 (2)
O3—Ni1—N5—Ni2	−6.86 (17)	N3—C22—C23—C24	67.2 (2)
O4—Ni2—N5—N6	147.40 (17)	N3—C22—C23—C25	−176.20 (16)
N3—Ni2—N5—N6	165.54 (19)	Ni2—N4—C24—C23	61.01 (19)
N8—Ni2—N5—N6	−29.93 (17)	C26—C23—C24—N4	59.6 (2)
N4—Ni2—N5—N6	−116.80 (17)	C25—C23—C24—N4	179.07 (17)
O5—Ni2—N5—N6	58.06 (17)	C22—C23—C24—N4	−64.4 (2)

### Hydrogen-bond geometry (Å, º)

**Table:**

*D*—H···*A*	*D*—H	H···*A*	*D*···*A*	*D*—H···*A*
O5—H5···O2	0.83 (1)	1.80 (1)	2.604 (2)	161 (3)
N2—H22···N10^i^	0.88 (1)	2.32 (2)	3.121 (2)	153 (2)
N4—H42···N10^i^	0.87 (1)	2.19 (1)	3.040 (2)	165 (2)

Symmetry code: (i) *x*+1, *y*, *z*.
